# The basal ganglia and the cerebellum in human emotion

**DOI:** 10.1093/scan/nsaa076

**Published:** 2020-06-08

**Authors:** Jordan E Pierce, Julie Péron

**Affiliations:** 1 Clinical and Experimental Neuropsychology Laboratory, University of Geneva, 1205 Geneva, Switzerland; 2 Neuropsychology Unit, Neurology Department, University Hospitals of Geneva, 1205 Geneva, Switzerland

**Keywords:** emotion, basal ganglia, cerebellum, learning, connectivity

## Abstract

The basal ganglia (BG) and the cerebellum historically have been relegated to a functional role in producing or modulating motor output. Recent research, however, has emphasized the importance of these subcortical structures in multiple functional domains, including affective processes such as emotion recognition, subjective feeling elicitation and reward valuation. The pathways through the thalamus that connect the BG and cerebellum directly to each other and with extensive regions of the cortex provide a structural basis for their combined influence on limbic function. By regulating cortical oscillations to guide learning and strengthening rewarded behaviors or thought patterns to achieve a desired goal state, these regions can shape the way an individual processes emotional stimuli. This review will discuss the basic structure and function of the BG and cerebellum and propose an updated view of their functional role in human affective processing.

## Introduction

The basal ganglia (BG) and the cerebellum traditionally have been assigned to roles within the motor domain, yet recent research has recognized their contributions to a variety of functions, including affective processing. Specifically, the roles of these subcortical structures have expanded beyond simple motor control to diverse limbic and cognitive processes including emotion recognition, reward- and error-based learning, language, decision-making, working memory and spatial attention (Haber and Knutson, 2010; Cavanagh *et al.*, 2011; Bostan *et al.*, 2013; Buckner, 2013; Péron *et al.*, 2017; Wojtecki *et al.*, 2017; Bostan and Strick, 2018; Eisinger *et al.*, 2018; Schmahmann, 2019). The current review will focus on the aspects of affective processing supported by each of these structures via their connections with a broad limbic network and on how recently identified direct subcortical connections between them may allow for coordinated modification of cortical responses to emotion in humans.

In general, limbic cortical and subcortical regions supporting emotion allow individuals to identify and prepare reactions to salient environmental stimuli, shaping how one perceives the world and interacts with positive or negative cues (Scherer, 2009; Brosch *et al.*, 2013). The amygdala has long been recognized as a central structure for the detection of salient emotional stimuli, modifying responses in sensory cortex to favor relevant items, recruiting top-down attention in frontal-parietal cortex, driving hypothalamic and brainstem autonomic reactions and biasing hippocampal memory formation (Sander *et al.*, 2003; Vuilleumier, 2005; Brosch *et al.*, 2013). Neocortical regions such as ventromedial and orbitofrontal cortex, anterior cingulate cortex and anterior insula also are involved in emotion and reward processing, further guiding the recognition of relevant sensory input, motivation for action and generation of an appropriate emotional response (Dolan, 2002; Thielscher and Pessoa, 2007; Haber and Knutson, 2010; Rudebeck and Rich, 2018).

Recent evidence from neuroimaging studies suggests that these core limbic regions are supported by the BG and cerebellum during affective tasks (Tanaka *et al.*, 2004; Baumann and Mattingley, 2012; Schraa-Tam *et al.*, 2012). Additionally, clinical assessments have highlighted the deficits in emotion processing following lesions to the BG or cerebellum (Schmahmann and Sherman, 1997; Le Jeune *et al.*, 2008; Péron *et al.*, 2017; Thomasson *et al.*, 2019), while structural and functional alterations have been reported in several psychiatric disorders (Gunaydin and Kreitzer, 2016; Lupo *et al.*, 2018). These findings support the idea that the BG and cerebellum contribute to a range of affective functions including recognition of emotional stimuli such as faces or voices, reward processing, generation of autonomic responses, inferring the emotional state of others (theory of mind) and awareness of subjective feelings (Clausi *et al.*, 2019b; Cox and Witten, 2019; Ferrari *et al.*, 2018; Péron *et al.*, 2010; Schmahmann, 2019; Strata, 2015).

**Fig. 1:**
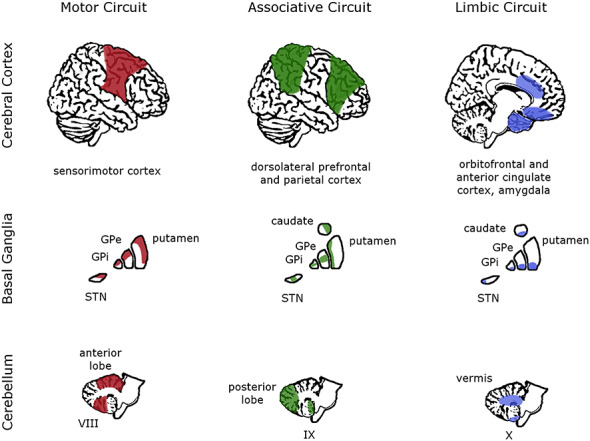
Motor, associative and limbic functional subdivisions in the cortex, BG and cerebellum. The colored portions of each brain region highlight the major areas associated with each general function, illustrating the most distinct connectivity of each subcortical subdivision with widespread cortical areas. The cerebral cortex is shown from a lateral and midsagittal view, the BG from a coronal view and the cerebellum from a midsagittal view. GPi/GPe, internal/external globus pallidus; STN, subthalamic nucleus; VIII, IX, X, cerebellar hemispheric lobules VIII, IX, X. Adapted from Krack *et al.* (2010) with permission.

Furthermore, connections exist between many parts of the limbic network (e.g. amygdala, orbitofrontal cortex) and the BG and cerebellum, which, in addition to the direct connection between these two regions, allow for a modulatory influence on affective processing across the brain (Hoshi *et al.*, 2005; Habas *et al.*, 2009; Lambert *et al.*, 2012). Historically, these multisynaptic connections between the BG and cerebellum, and with the neocortex, have been difficult to identify, but newer viral tracing procedures have allowed researchers to isolate their input/output channels in animal studies (Hoshi *et al.*, 2005; Bostan *et al.*, 2010). In humans, advances in neuroimaging techniques have provided evidence of similar connectivity between the BG, cerebellum and cortex (Habas *et al.*, 2009; Pelzer *et al.*, 2013; Milardi *et al.*, 2016). The BG and cerebellum, indeed, are connected structurally and functionally with most of the neocortex and contain sensorimotor, associative and limbic functional domains organized in multiple input/output loops (Figure 1; Buckner *et al.*, 2011; Mathai and Smith, 2011). The highly connected nature of the BG and cerebellum (Hoshi *et al.*, 2005; Bostan and Strick, 2018) allows them to perform parallel and integrated processing of cortical inputs to select and modify behavioral response patterns, resulting in a broad influence on emotion, informed by higher cognition, and with ready access to the motor network. The following sections of this review will describe the anatomy and function of the BG and cerebellum in relation to emotion processing, focusing primarily on human studies from healthy individuals as well as clinical populations, before highlighting their direct connectivity and proposed combined influence on cortical affective responses.

## Basic anatomy and function of the BG

The BG are a set of subcortical nuclei including the striatum (caudate/putamen/nucleus accumbens), globus pallidus (internal/external segments), substantia nigra and subthalamic nucleus (STN). The primary input nucleus in the BG is the striatum, which receives excitatory input from most of the cortex (Mink, 1996), the thalamus (Nambu *et al.*, 2002) and the deep cerebellar nuclei (DCN) (as discussed below; Hoshi *et al.*, 2005; Chen *et al.*, 2014; Xiao *et al.*, 2018). Cortical input to the striatum is organized according to functional domains, in broadly topographic stripes, with sensorimotor, associative and limbic subdivisions (Parent and Hazrati, 1995a; Mink, 1996; Arsalidou *et al.*, 2013). The striatum then sends inhibitory projections to other BG nuclei via the direct and indirect pathways, which largely maintain these functional subdivisions, ultimately shaping the appropriate behavioral response in cortical output regions via decreased or increased inhibition of thalamocortical pathways, respectively (Parent and Hazrati, 1995a; Lanciego *et al.*, 2012; Simonyan, 2019).

### Affective functions of the striatum and STN

Cortical input to the striatum is supplemented by motivation- and reward-sensitive dopamine release from the substantia nigra pars compacta and the ventral tegmental area in the midbrain (Jin *et al.*, 2014; Keeler *et al.*, 2014; Ikemoto *et al.*, 2015; Bostan and Strick, 2018). These reward signals support reinforcement learning (Box 1) in the BG, with positive or negative feedback to a behavior or internal state shaping its affective value and how the individual will respond to future occurrences of a given condition (Schultz *et al.*, 1997; Caligiore *et al.*, 2019). Dopaminergic neurons encode the reward signal as a prediction error, representing the difference between the expected reward and the actual reward received, meaning that unexpected rewards result in the largest responses because they are the most informative for updating values associated with a stimulus or condition (Schultz *et al.*, 1997; Daniel and Pollmann, 2014).

Box 1: Learning in the cortex, BG and cerebellum

Current models of learning posit distinct mechanisms in different regions of the brain: unsupervised learning occurs in the cortex, supervised learning occurs in the cerebellum, and reinforcement learning occurs in the BG (Doya, 2000; Bostan and Strick, 2018). Unsupervised learning refers to strengthening the associations between stimuli and/or responses based on simultaneous neuronal firing (Hebbian plasticity; Markram *et al.*, 1997). Supervised learning refers to improving performance based on minimization of prediction error signals (Wolpert *et al.*, 1998; Peterburs and Desmond, 2016). Finally, reinforcement learning refers to increasing the likelihood of a behavior based on a history of positive reward (or decreasing the likelihood of a punished behavior; trial and error learning (Doya, 2000; Caligiore *et al.*, 2019)). These learning mechanisms undergo complex transformations across the life span and interact as the various brain regions work collectively to achieve optimal performance in a given context (Caligiore *et al.*, 2019). All three systems require coordinated interactions to generate normal functioning for ongoing behavior. It remains undetermined, however, whether these models based on motor learning apply to affective processing. Presumably, the limbic domain within the BG and cerebellum functions in a similar manner to the motor domain but utilizes its connections with limbic regions to impact emotion processing.

Even with these unique functional connections, however, it may seem that rapid emotional responses would not rely heavily on slower learning processes. Nonetheless, even the simple case of viewing an emotional face could initiate learning in these subcortical regions. Emotion recognition could recruit the BG to process how the face or the individual’s response was rewarded and recruit the cerebellum to adjust the response based on how the individual’s current state matches a prediction for the specific emotional context. These modifications may be relevant particularly when the individual’s response to the emotional face is unexpectedly rewarded or punished, generating a large prediction error and strong dopamine release (Schultz *et al.*, 2000; K. S. Wang *et al.*, 2016; Yin *et al.*, 2008). The violation of the cerebellum’s internal model prediction would drive strong output signals to the cortex to correct the behavior according to the new reward signals from the BG. Together, the BG and cerebellum could synchronize and modulate activity in the amygdala, orbitofrontal cortex and visual cortex that corresponds to the appropriate emotional response and strengthen this response pattern for future appearances of such a stimulus (Péron *et al.*, 2013). The specifics of these learning processes remain to be clarified, however, and future research could manipulate rewards or prediction errors in emotion recognition tasks to elucidate how the different learning mechanisms contribute to affective processing.

Furthermore, the size of the reward drives the neural response within the ventral portion of the striatum, with the nucleus accumbens showing a greater response to large rewards of various types (e.g. food, money (Knutson *et al.*, 2005; Haber and Knutson, 2010), as well as to punishments (Shigemune *et al.*, 2013). The dorsal striatum, on the other hand, may be more sensitive to motivation (Miller *et al.*, 2014) and instrumental learning of action–outcome contingencies (Delgado, 2007; Yin *et al.*, 2008). This reward valuation process is related closely to the appraisal of the emotional valence of the stimulus with relevant rewarding stimuli tending to elicit positive emotions and motivate approach behaviors (Brosch *et al.*, 2010; Barbaro *et al.*, 2017; Sander *et al.*, 2018), although the causality and timing of these factors are complex and context-dependent (Grandjean and Scherer, 2008; Paul *et al.*, 2020). Overall, the stimulation of dopamine-sensitive neurons across the striatum results in increased activity in the direct pathway and decreased activity in the indirect pathway, further strengthening a selected behavior following a positive outcome (Kravitz *et al.*, 2012; Calabresi *et al.*, 2014).

Another important region of the BG in affective processing is the STN, which receives input from other BG nuclei as well as (frontal) cortex, the posterior thalamus (distinct from projections to the striatum) and the amygdala (Parent and Hazrati, 1995b; Nambu *et al.*, 2002; Lambert *et al.*, 2012; Péron *et al.,* 2016). These broad connections and findings of limbic and associative functional subdivisions within the STN indicate a critical role in emotional processing (Kuhn *et al.*, 2005; Temel *et al.*, 2005; Brunenberg *et al.*, 2012; Haynes and Haber, 2013; Péron *et al.*, 2013; Rossi *et al.*, 2015; Sieger *et al.*, 2015; Péron *et al.*, 2016). It has been proposed that the STN is responsible for integrating input across functional domains to modulate neural oscillations in cortical output regions, affecting multiple facets of emotion processing from autonomic arousal to emotional motor expression to subjective feeling (Péron *et al.*, 2013). Additionally, the hyperdirect pathway from the cortex through the STN may have a supervisory role in decision-making, establishing a threshold for action and inhibiting irrelevant activity based on salient contextual information provided by the prefrontal cortex (Cavanagh *et al.*, 2011; Haynes and Haber, 2013; Eisinger *et al.*, 2018).

### Insight from patient studies

While healthy functioning of the BG leads to behavioral optimization based on a history of rewarding outcomes, dysfunction of the dopamine-sensitive neurons in the ventral striatum (Robbins and Everitt, 1996; Schultz *et al.*, 2000; Haber and Knutson, 2010; Ikemoto *et al.*, 2015; Wang *et al.*, 2016) factors strongly into the occurrence of addiction disorders and substance abuse (Belin *et al.*, 2009; Calabresi *et al.*, 2014; Cox *et al.*, 2009; Makris *et al.*, 2008). The influence of dopamine on the BG results in the reinforcement of the initial behavior or internal state, but when these striatal reward processes are imbalanced, it can lead to repetitive pleasure-seeking behaviors that are dissociated from positive emotional feelings, as in cocaine dependency or gambling addiction (Belin and Everitt, 2008; Contreras-Rodriguez *et al.*, 2015; Luijten *et al.*, 2017). Furthermore, connections with the ventromedial prefrontal cortex, the anterior cingulate cortex, the hypothalamus and the amygdala allow the striatum to influence limbic processing in diverse contexts associated with reward valuation (Friedman *et al.*, 2002; Heimer and Van Hoesen, 2006; Haber, 2008; Ahmad *et al.*, 2017; Luijten *et al.*, 2017) and in psychiatric disorders such as depression and anxiety (Denys *et al.*, 2010; Robinson *et al.*, 2012; Ubl *et al.*, 2015; Gunaydin and Kreitzer, 2016).

Another condition caused by dysfunction of the BG is Parkinson’s disease, where a loss of dopaminergic neurons in the substantia nigra leads to motor deficits such as tremor. Importantly, however, the increasing use of deep brain stimulation (DBS) of the STN as a treatment for Parkinson’s disease has revealed the non-motor role of this structure as well, based on observations of cognitive and emotional side effects in some patients (Temel *et al.*, 2006; Halpern *et al.*, 2009; Zangaglia *et al.*, 2009; Rossi *et al.*, 2015; Mehanna *et al.*, 2017). Although the target of the stimulation is the STN motor domain, side effects include disruptions of verbal fluency (Anzak *et al.*, 2011; Eisinger *et al.*, 2018; B. Wu *et al.*, 2014), emotional facial recognition (Drapier *et al.*, 2008; Le Jeune *et al.*, 2008), auditory emotion recognition (Péron *et al.*, 2010), emotional conflict control (Irmen *et al.*, 2017) and subjective emotion experience (Vicente *et al.*, 2009).

For example, Drapier *et al.* (2008) tested Parkinson’s disease patients on measures of apathy and recognition of basic facial emotions 3 months before and after STN DBS surgery. They found that patients were significantly worse at recognizing fearful and sad faces and exhibited greater apathy following DBS treatment, although the two effects were not correlated. These results supported previous findings on the role of the STN in limbic facial recognition, potentially mediated by connections with the amygdala, orbitofrontal cortex and anterior cingulate cortex (Schroeder *et al.*, 2004; Biseul *et al.*, 2005; Drapier *et al.*, 2008; Le Jeune *et al.*, 2008; for a recent review, see Wagenbreth *et al.*, 2019). Similar impairments with negative emotions were described by Vicente *et al.* (2009) when asking STN DBS patients to report their subjective feelings in response to emotional film clips: the post-operative patients experienced diminished emotional responses to fearful and sad clips compared to the pre-operative and control groups. These effects may reflect a disruption of the STN’s ability to bias an appropriate response pattern, introducing noise into limbic processing and creating ambiguity between negative affective states that may share some underlying (e.g. physiological) features.

Other findings additionally suggest that STN stimulation in Parkinson’s disease can lead to impulsivity disorders or depressive symptoms (Volkmann *et al.*, 2001; Volkmann *et al.*, 2010; Moum *et al.*, 2012; Rossi *et al.*, 2015). In one study, 25% of Parkinson’s patients experienced depressive symptoms following STN DBS surgery (Berney *et al.*, 2002), although reports of mood changes vary widely and may depend on electrode placement or dopamine levels (Volkmann *et al.*, 2010). Interestingly, the impact of STN DBS on emotion or mood may depend upon the laterality of the substantia nigra degeneration in Parkinson’s disease: patients with predominantly left-sided motor impairments (right BG dysfunction) showed worse vocal emotion recognition for happy stimuli than patients with right-sided impairments (left BG dysfunction) or controls (Stirnimann *et al.*, 2018). These results imply that the right BG play a greater role in affective processes (Péron *et al.*, 2017), but further research is needed to confirm these findings and understand how interactions with both the cortex and cerebellum impact this laterality.

In addition to deficits in emotional behavior performance, Parkinson’s disease patients also have exhibited changes in glucose metabolism in emotion networks throughout the brain, including the orbitofrontal cortex (Le Jeune *et al.*, 2008; Péron *et al.*, 2013; Ory *et al.*, 2017) and the cerebellum. An increased cerebellar metabolism was associated with worse facial emotion recognition (Le Jeune *et al.*, 2008) and a decreased subjective feeling of disgust after viewing emotional film clips (Ory *et al.*, 2017). Such widespread changes support the functional link between the BG and cerebellum (see Section 5) and imply broad effects on emotion processing when STN function is disrupted. It is important to note, however, that these results partially may reflect pathological brain function, dopaminergic medication effects or other non-specific effects of the DBS procedure.

### The BG select and coordinate cortical response patterns

The studies described above demonstrate that the BG support not only motor functions (Graybiel, 1998; Yin *et al.*, 2004; Keeler *et al.*, 2014; Pidoux *et al.*, 2018) but also limbic functions such as reward valuation and motivation (Yin *et al.*, 2008; Miller *et al.*, 2014) that contribute to the assessment of affective valence and formation of emotional states. Clinical reports highlight the key function of the striatum in reward processing in addiction and psychiatric disorders and the contribution of the STN to emotion recognition. Overall, the dense connections and functional organizations of the BG position them perfectly to exert a broad coordinating influence on emotion processing, a function that is supported further by their interactions with the cerebellum.

## Basic anatomy and function of the cerebellum

The cerebellum contains more neurons than the neocortex, yet historically has been relegated to a purely motor role, perhaps due in part to its homogeneous cellular architecture arguing against a complex functional role in limbic or cognitive processing (Schmahmann, 2019). Nonetheless, recent research has reenergized interest in this structure and illuminated its contributions to diverse processes, including those arising from connections with the BG (discussed in the following section). Here, the intrinsic organization of the cerebellum and its numerous connections are described in relation to our growing understanding of its functional role in emotion processing.

The cerebellum consists of the vermis, paravermis, hemispheric lobules I–X and the DCN (consisting of the dentate, emboliform/globose (interpositus) and fastigial nuclei). It receives input from the inferior olivary complex and other brainstem areas, as well as from pontine nuclei that transmit signals from the neocortex (Kelly and Strick, 2003; Granziera *et al.*, 2009). The output of the cerebellum occurs via inhibitory Purkinje cells, which project to the DCN and then to the thalamus and ultimately feedback to the same regions of the cortex from which the cerebellum receives input. The multisynaptic nature of these connections has made them difficult to identify until recently, but it is recognized now that nearly all of cortex, not merely motor areas, projects to and receives projections from the cerebellum (Middleton and Strick, 1994; Kelly and Strick, 2003; Bostan *et al.*, 2013; Buckner, 2013).

The cerebellum also has functional subdivisions, similar to those identified in the BG: motor, association and limbic zones with broadly topographic organization (Leggio and Olivito, 2018; Schmahmann, 2019). The anterior lobules are connected primarily with sensorimotor cortical and brainstem regions, the posterior lobules are connected heavily with cortical association regions, and parts of the vermis and flocculonodular lobe are connected via the fastigial nucleus to limbic cortical and subcortical regions (Figure 1; Anand *et al.*, 1959; Heath and Harper, 1974; Olivito *et al.*, 2017a; Schmahmann, 2019). The anterior lobe mainly performs the traditional motor coordination role attributed to the cerebellum, yet the association regions in the posterior lobe constitute the greatest proportion of cerebellar volume in humans, reflecting an evolutionary expansion in attention and executive functions in frontal and parietal cortices (Schmahmann, 2019). Furthermore, these functional domains may be maintained in the DCN, with studies identifying both motor and non-motor projections of the dentate nucleus in particular (Dum and Strick, 2003; Kuper *et al.*, 2011; Bostan *et al.*, 2013).

In the limbic cerebellum, the vermis has been associated with basic emotions such as fear, while regions of the posterior cerebellar hemispheres have been associated with complex emotions and social interactions, reflecting the former’s anatomical connections with brainstem nuclei controlling autonomic functions and the latter’s connections with associative prefrontal cortex controlling theory of mind and higher cognitive functions (Stoodley and Schmahmann, 2009; Watson *et al.*, 2013; Van Overwalle *et al.,* 2014; Strata, 2015; Leggio and Olivito, 2018). Rodent studies have demonstrated that the limbic cerebellum and its connections with the amygdala contribute to fear-related learning (Supple Jr. *et al.*, 1987; Sacchetti *et al.*, 2002; Zhu *et al.*, 2011; Strata, 2015), while human studies (described below) have indicated activity in the vermis in response to both negative and positive emotions (Baumann and Mattingley, 2012; Schraa-Tam *et al.*, 2012).

### Neuroimaging studies on the affective functions of the cerebellum

The use of neuroimaging and sensitive neurological assessments in recent years has vastly expanded our understanding of the human cerebellum’s role in a wide range of non-motor functions (Koziol *et al.*, 2014). Early studies showed cerebellar activation during a word generation task (Petersen *et al.*, 1989) and a problem-solving task (Kim *et al.*, 1994), while more recent reviews and meta-analyses have highlighted the cerebellum’s functional involvement in diverse tasks related to emotion, attention, working memory and language (Buckner, 2013; Adamaszek *et al.*, 2017; Guell *et al.*, 2018; Pleger and Timmann, 2018; Stoodley and Schmahmann, 2018; Schmahmann, 2019). Additionally, resting-state neuroimaging studies have reported cerebellar co-activation with common cortical functional networks, and noted an expanded cerebellar representation of frontal-parietal association areas (Buckner *et al.*, 2011; Marek *et al.*, 2018), as well as co-activation with limbic networks that included the amygdala, insula and BG (Sang *et al.*, 2012; Habas, 2018).

Neuroimaging studies of healthy individuals recently have demonstrated the relevance of the cerebellum (particularly the vermis, hemispheric Crus I and II and the fastigial nucleus) to emotion perception and recognition (Lee *et al.*, 2004; Bermpohl *et al.*, 2006; Stoodley and Schmahmann, 2009; Baumann and Mattingley, 2012; Schraa-Tam *et al.*, 2012; Adamaszek *et al.*, 2017; Ferrari *et al.*, 2018). Baumann and Mattingley (2012) proposed that the vermis, as part of the limbic cerebellum, assesses emotional relevance (threat) and regulates emotional responses, supported by connections with the medial prefrontal cortex and the amygdala. Additionally, they reported partially distinct cerebellar activation patterns for individual emotion categories with significant activation for both negative and positive emotions (Baumann and Mattingley, 2012), although others have reported activation primarily for negative emotions (Beauregard *et al.*, 1998; Schraa-Tam *et al.*, 2012; Adamaszek *et al.*, 2017). These results are complemented by functional connectivity studies and meta-analyses of task data that have identified co-activation of widespread emotion networks with portions of the cerebellum (Habas *et al.*, 2009; Stoodley and Schmahmann, 2009; Schienle and Scharmuller, 2013; Guell *et al.*, 2018; Habas, 2018).

Interestingly, some findings demonstrated a lateralization of cerebellum function with greater language activation in the right cerebellum and greater spatial attention activation in the left cerebellum (Petersen *et al.*, 1989; Habas *et al.*, 2009; Lesage *et al.*, 2012; Wang *et al.*, 2013; Schmahmann, 2019). This cerebellar lateralization mirrors neocortical lateralization, reflecting the contralateral anatomical connectivity between, e.g. left frontal cortex language centers and right cerebellar cortex (Connor *et al.*, 2006; Frings *et al.*, 2006; Kelly and Strick, 2003; D. Wang *et al.*, 2013; Xiao *et al.*, 2018).

While neuroimaging studies describe functional activation correlated with task performance, neuromodulatory studies seek to assess the causal impact of the cerebellum on emotion processing by directly affecting neural activity. In a study by Schutter and van Honk, repetitive transcranial magnetic stimulation (TMS) was applied to the cerebellar vermis and occipital cortex of healthy subjects prior to an emotion regulation task (Schutter and van Honk, 2009). Following inhibition of only the cerebellum, an increase in negative mood was reported after viewing aversive scenes, suggesting a disruption of the limbic network’s ability to regulate or suppress negative affect. In another study, high-frequency, excitatory TMS of the medial cerebellum resulted in increased reaction times for an implicit processing of masked happy faces, suggesting enhanced attention to these emotional stimuli (Schutter *et al.*, 2009). Further work utilizing transcranial direct current stimulation of the cerebellum also reported improved processing of negative (but not positive) facial expressions (Ferrucci *et al.*, 2012). The differences in positive *vs* negative emotion effects remain to be clarified, although the specific region of the cerebellum that is targeted, the task design and the modulation strength may all contribute to the pattern of results in these stimulation studies.

Many human studies on the cerebellum have focused on basic emotion recognition, yet the cerebellum also has been shown to be involved in reward processing in relation to prediction error. Studies in mice have demonstrated that climbing fibers and granule cells signal reward occurrence, omission or predictability to functional microzones of Purkinje cells (Wagner *et al.*, 2017; Kostadinov *et al.*, 2019), while reward-related activations also have been reported in human neuroimaging studies (Ramnani *et al.*, 2004; Tanaka *et al.*, 2004; Shigemune *et al.*, 2013), likely in relation to updating of internal models (Peterburs *et al.*, 2018). Furthermore, the cerebellum has been shown to have direct connections with the dopaminergic ventral tegmental area and BG nuclei (Ikai *et al.*, 1992; Herrera-Meza *et al.*, 2014; Carta *et al.*, 2019), providing a further link with the reward system and affective valuation.

### Insight from patient studies

Beyond neuroimaging studies of healthy cerebellar function, studies of cerebellar lesion patients have elucidated the role of this structure in affective processes by identifying specific behavioral difficulties that occur in patients (Schmahmann, 2019). Reports have described problems with emotion recognition and expression in patients, as well as alterations in mood control and the experience of emotional states, with cerebellar patients exhibiting a variety of behavioral and mood disturbances (Schmahmann *et al.*, 2007; Lupo *et al.*, 2015; Clausi *et al.*, 2019a). Problems with social cognition and understanding of others’ emotions have been reported in patients with cerebellar degeneration, including spinocerebellar ataxia, potentially related to disrupted formation of internal models that predict others’ behavior using theory of mind processes (Sokolovsky *et al.*, 2010; Leggio and Olivito, 2018; Clausi *et al.*, 2019b). More generally, damage to or reduced volume of the cerebellum is associated with high rates of depression, anxiety, psychosis and autism spectrum disorders, and patients may experience blunted affect, uncontrollable laughter or crying or increased aggression (Escalona *et al.*, 1993; Leroi *et al.*, 2002; Schmahmann *et al.*, 2007; Schutter *et al.*, 2012; Lupo *et al.*, 2018).

Considering autism spectrum disorders in more detail, difficulties with social interaction and emotion recognition are common, as are cerebellar (and BG) structural abnormalities including a reduction in the number of Purkinje cells in the cerebellar hemispheres (Whitney *et al.*, 2008; Fatemi *et al.*, 2012; Subramanian *et al.*, 2017; Bruchhage *et al.*, 2018). Reduced volume of the posterior vermis also is associated with autism spectrum disorders, although a variety of malformations, both within and beyond the cerebellum, have been reported (Scott *et al.*, 2009; Bruchhage *et al.*, 2018). The exact role of cerebellar dysfunction in autism and other disorders remains to be defined fully, as even a focal lesion may lead to network level changes in structure or function over time (Hernandez-Castillo *et al.*, 2015; Olivito *et al.*, 2017a; Olivito *et al.*, 2017b), but it is clear that some association exists between cerebellar abnormalities and social, emotional and cognitive dysfunctions in several conditions (Bruchhage *et al.*, 2018; Olivito *et al.*, 2018; Schmahmann, 2019). Finally, such clinical symptoms may depend upon the functional connections of the cerebellum with the BG and limbic circuitry as it has been suggested that hyperactivation of the cerebellum in Parkinson’s disease results from the decreased activity of BG dopaminergic nuclei (T. Wu and Hallett, 2005; Yu *et al.*, 2007).

Beyond these assessments of distinct clinical disorders, other studies have addressed more subtle alterations in affective performance in patients with cerebellar damage. One study found that cerebellar patients experienced weaker positive emotion than controls when viewing pleasant images and that they differentially recruited an emotion network (greater prefrontal/BG activation and weaker amygdala activation) when viewing unpleasant images (Turner *et al.*, 2007). Another study showed that cerebellar patients had impaired awareness of their own negative affect when it occurred as a result of their own actions (i.e. regret) in a gambling task (Clausi *et al.*, 2015). Finally, a recent study reported that cerebellar patients ranked fear stimuli higher on a surprise index than did control subjects and that this misattribution was associated with right-sided lesions in cerebellar hemisphere lobules VII, VIII and IX (Thomasson *et al.*, 2019; see also Biseul *et al.*, 2005 for a similar misattribution in STN DBS patients).

### The cerebellum minimizes prediction error across domains

Based on the variety of tasks that activate the cerebellum and its largely uniform neuronal architecture, Schmahmann has proposed that the function of the cerebellum is comparable across motor, cognitive and limbic domains, performing an automatic modulation of processing based on the widespread inputs it receives about ongoing behaviors or thoughts compared to the desired or predicted outcome (Schmahmann, 1991, 2019). Subsequently, the cerebellum provides feedback to the cortex for fine-tuning not only motor output but also processes such as decision-making or emotion recognition (Bostan and Strick, 2018; Schmahmann, 2019). Similar theories emphasize the cerebellum’s role in detecting and minimizing prediction error based on differences in current sensorimotor and cognitive information and the intended goal state (referred to as supervised learning; see Box 1; Jueptner and Weiller, 1998; Sokolov *et al.*, 2017; Caligiore *et al.*, 2019; Popa and Ebner, 2019).

Such a broad function in monitoring the current state relative to a desired or expected state in order to adjust behavior is consistent with the cerebellum’s closed loop connectivity with the cortex (Kelly and Strick, 2003; Bostan and Strick, 2018) and with the difficulty in pinpointing a single cognitive dysfunction across patients with cerebellar lesions (Schmahmann and Sherman, 1997; Schmahmann *et al.*, 2007; Lupo *et al.*, 2018; Schmahmann, 2019). Combining this understanding of cerebellar anatomy and function with that of the BG, the next sections will examine evidence of the structural connectivity between these subcortical regions and how this allows for a coordinated influence on affective processing.

## Direct connectivity between the BG and cerebellum

**Fig. 2:**
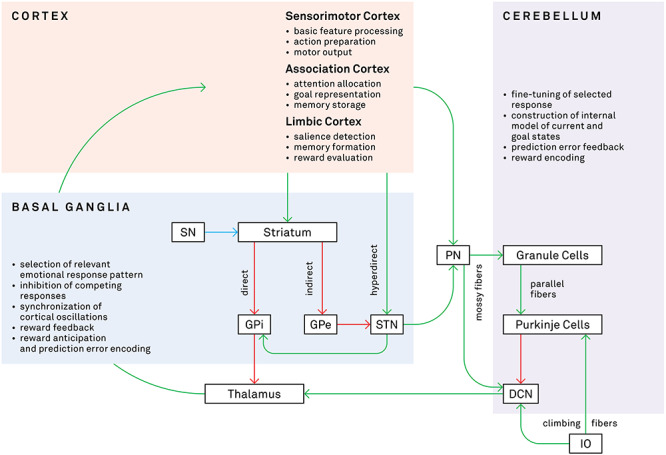
Connections and select functions of the BG, cerebellum and cortex. This diagram shows the structural organization and functions of relevant pathways in the BG, cerebellum and cortex. During processing of an emotional stimulus, these regions (as well as, e.g. amygdala, hippocampus) cooperatively analyze the incoming information and generate an appropriate response, which includes action tendencies, physiological changes and subjective feelings. Green arrows indicate excitatory pathways, red arrows indicate inhibitory pathways, and the blue arrow indicates the dopaminergic pathway from the SN. SN, substantia nigra; GPi/GPe, internal/external globus pallidus; STN, subthalamic nucleus; PN, pontine nuclei; DCN, deep cerebellar nuclei; IO, inferior olive.

It was initially assumed that the BG and cerebellum separately impacted cortical activity through distinct thalamic pathways (Mink, 1996; Middleton and Strick, 2000), yet the existence of direct subcortical connections between these two structures is now recognized and, thus, their ability to jointly influence motor, cognitive and limbic functions (Figure 2; Caligiore *et al.*, 2017; Bostan and Strick, 2018). The first study to identify a structural link between the BG and cerebellum in non-human primates used transneuronal viral tracing to label neurons in the dentate nucleus that project to the intralaminar nuclei of the thalamus and then to the striatum and the external globus pallidus (Hoshi *et al.*, 2005). Later work expanded this finding by showing a connection in the opposite direction, with the STN projecting to the pontine nuclei and then to the contralateral cerebellar hemisphere (Bostan *et al.*, 2010). In both cases, these projections innervated the motor and non-motor functional domains, meaning the BG and cerebellum can modulate a wide range of habitual and goal-directed behaviors (Xiao *et al.*, 2018). Furthermore, it has been shown that these connections exhibit short latency responses which would allow for rapid communication to impact early stages of processing (Chen *et al.*, 2014).

Importantly, the existence of these connections in humans has been supported by diffusion-weighted imaging studies, which can measure the strength of white matter tracts non-invasively. Pelzer et al. identified tracts between the dentate and striatum and the STN and cerebellum via the thalamus and pontine nuclei (Pelzer *et al.*, 2013; Pelzer *et al.*, 2017), mirroring the results from the earlier animal studies. Another study in humans extended these findings by reporting structural connections between the dentate and globus pallidus and substantia nigra (Milardi *et al.*, 2016). Functional connectivity studies also have confirmed the co-activation of BG nuclei and the cerebellum and associated these subcortical structures with multiple resting-state networks that encompass large portions of the neocortex, including limbic regions such as the orbitofrontal cortex and the amygdala (Habas *et al.*, 2009; Brunenberg *et al.*, 2012).

**Fig. 3:**
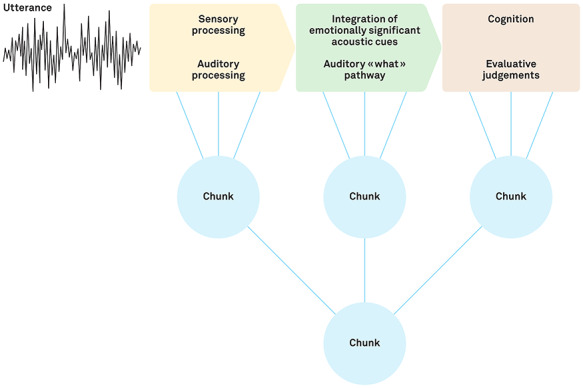
Example of the chunking process in the limbic domain using vocal emotion processing. As proposed by Schirmer and Kotz (2006), processing of an emotional utterance proceeds in three steps from primary auditory cortex to superior temporal cortex to inferior frontal cortex. Simultaneously, connections with the BG allow them to iteratively assess contexts and select actions to form or control the expression of emotional (or motor/cognitive) sequences, explaining the BG’s sensitivity to the temporal and structural organization of events. When the emotional sequences are recurrent, the BG creates units of these sequence representations called ‘chunks’ at each level of the limbic auditory processing stream. These smaller chunks then can be combined into a larger representative chunk for a single sequenced response to a given stimulus that is activated without the need for attentional control of each step. Meanwhile, the cerebellum monitors prediction errors of internal models relying upon the performance of these chunks and can trigger adjustments to the ongoing sequenced behavior and recruit controlled processing as needed for optimal responding. Adapted with permission from Schirmer and Kotz (2006).

## Combined functional role of the BG and cerebellum in emotion

The direct connections between the BG and cerebellum allow these regions to work together to modulate processes such as motor control and emotion recognition or expression, with the two regions guiding the selection and precision of behavioral output. Both structures participate in learning appropriate responses (Box 1), primarily using reward feedback in the BG and prediction error feedback in the cerebellum to adjust response selection and execution (Doya, 2000; Tanaka *et al.*, 2004; Bostan *et al.*, 2013; Taylor and Ivry, 2014; Fermin *et al.*, 2016; Bostan and Strick, 2018; Eisinger *et al.*, 2018; Caligiore *et al.*, 2019). Importantly, their functions are not entirely distinct, however, but overlapping, with the BG also encoding prediction error (Cox and Witten, 2019; Pine *et al.*, 2018) and the cerebellum responding to reward presentation (Kostadinov *et al.*, 2019). The reward-related BG circuitry with differential dopaminergic sensitivity in the direct and indirect pathways allows for the selection and strengthening of an appropriate cortical response and inhibition of other, unrewarding responses. In conjunction with this selection process, the cerebellum’s uniform cellular architecture performs fine-tuning of the selected response (whether it be an emotional reaction, linguistic rehearsal or motor movement) to optimize the outcome based on the internal model of the individual’s current state.

### Subcortical synchronization and modulation of cortical emotional responses

The ventromedial STN, based on its advantageous position in the hyperdirect pathway consisting of closed loop connections with limbic frontal cortex, may be critical for (de)synchronizing cortical neural oscillations that produce an emotion response pattern following a salient environmental cue (Péron *et al.*, 2013). The BG’s sensitivity to the temporal characteristics of a stimulus (Buhusi and Meck, 2005; Péron *et al.*, 2017) allows for precise control of distant cortical activity with the inhibitory effect of increased STN output modulating cortical responses to emotional stimuli to generate an appropriate response while limiting competing signals from task-irrelevant or task-incidental co-activations. This modulatory effect on cortical targets is influenced further by dopaminergic input to the BG, which increases cortical excitability following rewarding stimuli or behaviors (Costa *et al.*, 2006).

By gating the starting and stopping of emotional response patterns (Ory *et al.*, 2017), the BG influence cortical learning as the relevant neural populations increase their connectivity strength through synchronous firing. Over time, repetition of this selection process leads to the creation of sequences of cortical representations that are ‘chunked’ together into habit-like responses that can occur nearly automatically following an emotional cue (as in the motor domain; Graybiel, 2008). As such, the BG may be particularly important during the acquisition of new emotional response patterns or in new emotional contexts when strong rewards or prediction errors lead to the formation or alteration of cortical response chunks. This chunking process allows for more rapid access to holistic sequenced responses without the need for costly attentional intervention, which is crucial when interacting with emotional stimuli such as a fearful facial expression.

The limbic cerebellum, on the other hand, modulates the amplitude of cortical oscillations based on prediction error feedback of the selected response relative to the given context (Booth *et al.*, 2007; Schmahmann, 2019). Input to the cerebellum regarding the salience and motivational value of emotional stimuli guides internal models to determine how an emotional response benefits individuals in their current state and, thus, shapes how output from the cerebellum modifies the limbic response pattern. By continuously monitoring performance of the individual in terms of prediction error, the cerebellum ensures that large deviations from the expected response/outcome are quickly corrected (Peterburs and Desmond, 2016). In cases of cerebellar lesions, however, a lack of cerebellar amplification of emotional networks may lead to the blunted affect and decreased subjective experience of emotions often reported in patients (Adamaszek *et al.*, 2017).

An example of the contribution of the BG and cerebellum to emotion processing can be found during perception of an angry voice (Figure 3). The recognition of relevant emotional prosody information begins in primary auditory cortex with extraction of basic acoustic features and then proceeds to more anterior regions of superior temporal cortex for identification of general emotional cues and then to the inferior frontal cortex for elaborated processing of the emotional salience and semantic content of the voice (Schirmer and Kotz, 2006). Throughout this processing pathway, the BG enhances activity within the neural representation (i.e. a habit-like ‘chunk’) corresponding to a previously reinforced experience with a similar angry voice, more quickly activating downstream regions to reach a decision threshold or generate a motor response that matches what was rewarded in the earlier encounter. Simultaneously, the cerebellum checks whether the state of the individual varies from the expected state at any time during the emotion recognition process. If this prediction error exceeds a given threshold defined by the context (e.g. the individual’s goals, familiarity with the speaker, stress levels), then the cerebellum can refine the cortical/BG response and recalibrate the internal model. Nonetheless, since the BG and cerebellum do not directly store or produce emotional response patterns themselves, lesions to these regions do not necessarily cause gross emotional deficits (but see Section 3.2 and Schmahmann and Sherman, 1998; Levisohn *et al.*, 2000, for significant clinical symptoms). Instead such damage leads to a more subtle neural miscoordination that may hinder efficient emotion recognition by introducing additional noise into the system (Péron *et al.*, 2013; Schmahmann, 2019).

Overall, in the healthy brain, repeated synchronization of cortical regions by the BG and fine-tuning by the cerebellum strengthen representations of a given response pattern and allow learned emotional responses to be generated more automatically in the future, just as with motor behaviors. Furthermore, studies suggest that this emotional synchronization occurs regardless of the valence (positive or negative) or modality (auditory or visual) of the emotional stimuli (Turner *et al.*, 2007; Baumann and Mattingley, 2012; Péron *et al.*, 2013, 2017), and during emotion recognition as well as production, assuming the limbic network reactivates in a similar fashion during perception without executing the motor expression of an emotional response (Ferrari *et al.*, 2018). Connections with regions controlling motivation (nucleus accumbens, substantia nigra, ventral tegmental area), relevance detection (amygdala) and physiological responses (brainstem) also imply a broad influence on emotion (Ikai *et al.*, 1992; Baumann and Mattingley, 2012; Péron *et al.*, 2013; Bostan and Strick, 2018; Habas, 2018) and warrant further attention in future neuroimaging studies.

The synchronization mechanism proposed above is consistent with the general function of the BG and cerebellum in other domains, suggesting that subdivisions of these structures perform similar processing in different tasks depending on the pattern of cortical and subcortical connectivities with different functional networks. Limbic, cognitive and motor functional domains in the BG and cerebellum could allow for integration of multiple cortical inputs within or across domains, perhaps occurring within the STN (Mathai and Smith, 2011; Péron *et al.*, 2013) and the DCN (Habas, 2010), respectively, as these small structures receive converging input from other parts of the BG or cerebellar hemispheres. Most evidence, however, indicates largely distinct processing streams for each functional domain that output to the same cortical regions from which they receive input (Middleton and Strick, 2000; Kuper *et al.*, 2011; Stoodley and Schmahmann, 2018). Additionally, different neural subpopulations could utilize distinct oscillation frequencies to gate activity for short- or long-range connections and to increase the computing power of limited neural resources. Ultimately, affective processing, like many other brain functions, relies on the selection and inhibition of response patterns by the BG and fine-tuning of the selected response by the cerebellum to minimize mismatch between one’s expected and actual internal state in a given context.

## Conclusion

Subcortical structures often have been ignored in the study of human cognition in favor of the larger and more accessible neocortex. Recent work, however, has highlighted the importance of the BG and cerebellum in emotion processing, which is mediated by their dense cortical and subcortical connections. Evidence for direct structural and functional connections between the BG and cerebellum further has demonstrated that these regions can influence brain activity both independently and through synchronization of widespread limbic brain regions to select and adjust responses based on the current contextual state, allowing for efficient acquisition and production of appropriate response patterns.

## Funding

This work was supported by the Swiss National Science Foundation (grant number 105314_182221 to J. P.).

## Conflict of interest

The authors declare that there are no financial conflicts of interest.

## References

[ref1] Adamaszek M., D’Agata F., Ferrucci R., et al. (2017). Consensus paper: cerebellum and emotion. The Cerebellum, 16(2), 552–76. doi: 10.1007/s12311-016-0815-8. 27485952

[ref2] Ahmad T., Sun N., Lyons D., Laviolette S.R. (2017). Bi-directional cannabinoid signalling in the basolateral amygdala controls rewarding and aversive emotional processing via functional regulation of the nucleus accumbens. Addiction Biology, 22(5), 1218–31. doi: 10.1111/adb.12406. 27230434

[ref3] Anand B.K., Malhotra C.L., Singh B., Dua S. (1959). Cerebellar projections to limbic system. Journal of Neurophysiology, 22(4), 451–7. doi: 10.1152/jn.1959.22.4.451. 13673296

[ref4] Anzak A., Gaynor L., Beigi M., et al. (2011). A gamma band specific role of the subthalamic nucleus in switching during verbal fluency tasks in Parkinson's disease. Experimental Neurology, 232(2), 136–42. doi: 10.1016/j.expneurol.2011.07.010. 21872587

[ref5] Arsalidou M., Duerden E.G., Taylor M.J. (2013). The Centre of the brain: topographical model of motor, cognitive, affective, and somatosensory functions of the basal ganglia. Human Brain Mapping, 34(11), 3031–54. doi: 10.1002/hbm.22124. 22711692 PMC6870003

[ref6] Barbaro L., Peelen M.V., Hickey C. (2017). Valence, not utility, underlies reward-driven prioritization in human vision. The Journal of Neuroscience, 37(43), 10438–50. doi: 10.1523/jneurosci.1128-17.2017. 28951452 PMC6596625

[ref7] Baumann O., Mattingley J.B. (2012). Functional topography of primary emotion processing in the human cerebellum. NeuroImage, 61(4), 805–11. doi: 10.1016/j.neuroimage.2012.03.044. 22465459

[ref8] Beauregard M., Leroux J.M., Bergman S., et al. (1998). The functional neuroanatomy of major depression: an fMRI study using an emotional activation paradigm. Neuroreport, 9(14), 3253–8. doi: 10.1097/00001756-199810050-00022. 9831460

[ref9] Belin D., Everitt B.J. (2008). Cocaine seeking habits depend upon dopamine-dependent serial connectivity linking the ventral with the dorsal striatum. Neuron, 57(3), 432–41. doi: 10.1016/j.neuron.2007.12.019. 18255035

[ref10] Belin D., Jonkman S., Dickinson A., Robbins T.W., Everitt B.J. (2009). Parallel and interactive learning processes within the basal ganglia: relevance for the understanding of addiction. Behavioural Brain Research, 199(1), 89–102. doi: 10.1016/j.bbr.2008.09.027. 18950658

[ref11] Bermpohl F., Pascual-Leone A., Amedi A., et al. (2006). Dissociable networks for the expectancy and perception of emotional stimuli in the human brain. NeuroImage, 30(2), 588–600. doi: 10.1016/j.neuroimage.2005.09.040. 16275018

[ref12] Berney A., Vingerhoets F., Perrin A., et al. (2002). Effect on mood of subthalamic DBS for Parkinson's disease: a consecutive series of 24 patients. Neurology, 59(9), 1427–9. doi: 10.1212/01.wnl.0000032756.14298.18. 12427897

[ref13] Biseul I., Sauleau P., Haegelen C., et al. (2005). Fear recognition is impaired by subthalamic nucleus stimulation in Parkinson's disease. Neuropsychologia, 43(7), 1054–9. doi: 10.1016/j.neuropsychologia.2004.10.006. 15769491

[ref14] Booth J.R., Wood L., Lu D., Houk J.C., Bitan T. (2007). The role of the basal ganglia and cerebellum in language processing. Brain Research, 1133, 136–44. doi: 10.1016/j.brainres.2006.11.074. 17189619 PMC2424405

[ref15] Bostan A.C., Strick P.L. (2018). The basal ganglia and the cerebellum: nodes in an integrated network. Nature Reviews. Neuroscience, 19(6), 338–50. doi: 10.1038/s41583-018-0002-7. 29643480 PMC6503669

[ref16] Bostan A.C., Dum R.P., Strick P.L. (2010). The basal ganglia communicate with the cerebellum. Proceedings of the National Academy of Sciences of the United States of America, 107(18), 8452–6. doi: 10.1073/pnas.1000496107. 20404184 PMC2889518

[ref17] Bostan A.C., Dum R.P., Strick P.L. (2013). Cerebellar networks with the cerebral cortex and basal ganglia. Trends in Cognitive Sciences, 17(5), 241–54. doi: 10.1016/j.tics.2013.03.003. 23579055 PMC3645327

[ref18] Brosch T., Pourtois G., Sander D. (2010). The perception and categorisation of emotional stimuli: a review. Cognition and Emotion, 24(3), 377–400. doi: 10.1080/02699930902975754.

[ref19] Brosch T., Scherer K.R., Grandjean D., Sander D. (2013). The impact of emotion on perception, attention, memory, and decision-making. Swiss Medical Weekly, 143, w13786. doi: 10.4414/smw.2013.13786. 23740562

[ref20] Bruchhage M.M.K., Bucci M.P., Becker E.B.E. (2018). Cerebellar involvement in autism and ADHD. Handbook of Clinical Neurology, 155, 61–72. doi: 10.1016/b978-0-444-64189-2.00004-4. 29891077

[ref21] Brunenberg E.J., Moeskops P., Backes W.H., et al. (2012). Structural and resting state functional connectivity of the subthalamic nucleus: identification of motor STN parts and the hyperdirect pathway. PLoS One, 7(6), e39061. doi: 10.1371/journal.pone.0039061. 22768059 PMC3387169

[ref22] Buckner R.L. (2013). The cerebellum and cognitive function: 25 years of insight from anatomy and neuroimaging. Neuron, 80(3), 807–15. doi: 10.1016/j.neuron.2013.10.044. 24183029

[ref23] Buckner R.L., Krienen F.M., Castellanos A., Diaz J.C., Yeo B.T. (2011). The organization of the human cerebellum estimated by intrinsic functional connectivity. Journal of Neurophysiology, 106(5), 2322–45. doi: 10.1152/jn.00339.2011. 21795627 PMC3214121

[ref24] Buhusi C.V., Meck W.H. (2005). What makes us tick? Functional and neural mechanisms of interval timing. Nature Reviews. Neuroscience, 6(10), 755–65. doi: 10.1038/nrn1764. 16163383

[ref25] Calabresi P., Picconi B., Tozzi A., Ghiglieri V., Di Filippo M. (2014). Direct and indirect pathways of basal ganglia: a critical reappraisal. Nature Neuroscience, 17(8), 1022–30. doi: 10.1038/nn.3743. 25065439

[ref26] Caligiore D., Pezzulo G., Baldassarre G., et al. (2017). Consensus paper: towards a systems-level view of cerebellar function: the interplay between cerebellum, basal ganglia, and cortex. The Cerebellum, 16(1), 203–29. doi: 10.1007/s12311-016-0763-3. 26873754 PMC5243918

[ref27] Caligiore D., Arbib M.A., Miall R.C., Baldassarre G. (2019). The super-learning hypothesis: integrating learning processes across cortex, cerebellum and basal ganglia. Neuroscience and Biobehavioral Reviews, 100, 19–34. doi: 10.1016/j.neubiorev.2019.02.008. 30790636

[ref28] Carta I., Chen C.H., Schott A.L., Dorizan S., Khodakhah K. (2019). Cerebellar modulation of the reward circuitry and social behavior. Science, 363(6424). doi: 10.1126/science.aav0581. PMC6711161 30655412

[ref29] Cavanagh J.F., Wiecki T.V., Cohen M.X., et al. (2011). Subthalamic nucleus stimulation reverses mediofrontal influence over decision threshold. Nature Neuroscience, 14, 1462. doi: 10.1038/nn.2925. 21946325 PMC3394226

[ref30] Chen C.H., Fremont R., Arteaga-Bracho E.E., Khodakhah K. (2014). Short latency cerebellar modulation of the basal ganglia. Nature Neuroscience, 17(12), 1767–75. doi: 10.1038/nn.3868. 25402853 PMC4241171

[ref31] Clausi S., Coricelli G., Pisotta I., et al. (2015). Cerebellar damage impairs the self-rating of regret feeling in a gambling task. Frontiers in Behavioral Neuroscience, 9(113), 1–10. doi: 10.3389/fnbeh.2015.00113. 25999829 PMC4419712

[ref32] Clausi S., Lupo M., Olivito G., et al. (2019a). Depression disorder in patients with cerebellar damage: awareness of the mood state. Journal of Affective Disorders, 245, 386–93. doi: 10.1016/j.jad.2018.11.029. 30423466

[ref33] Clausi S., Olivito G., Lupo M., Siciliano L., Bozzali M., Leggio M. (2019b). The cerebellar predictions for social interactions: theory of mind abilities in patients with degenerative cerebellar atrophy. Frontiers in Cellular Neuroscience, 12, 510. doi: 10.3389/fncel.2018.00510. 30670949 PMC6332472

[ref34] Connor L.T., DeShazo Braby T., Snyder A.Z., Lewis C., Blasi V., Corbetta M. (2006). Cerebellar activity switches hemispheres with cerebral recovery in aphasia. Neuropsychologia, 44(2), 171–7. doi: 10.1016/j.neuropsychologia.2005.05.019. 16019040

[ref35] Contreras-Rodriguez O., Albein-Urios N., Perales J.C., et al. (2015). Cocaine-specific neuroplasticity in the ventral striatum network is linked to delay discounting and drug relapse. Addiction, 110(12), 1953–62. doi: 10.1111/add.13076. 26212416

[ref36] Costa R.M., Lin S.C., Sotnikova T.D., et al. (2006). Rapid alterations in corticostriatal ensemble coordination during acute dopamine-dependent motor dysfunction. Neuron, 52(2), 359–69. doi: 10.1016/j.neuron.2006.07.030. 17046697

[ref37] Cox J., Witten I.B. (2019). Striatal circuits for reward learning and decision-making. Nature Reviews. Neuroscience, 20(8), 482–94. doi: 10.1038/s41583-019-0189-2. 31171839 PMC7231228

[ref38] Cox S.M., Benkelfat C., Dagher A., et al. (2009). Striatal dopamine responses to intranasal cocaine self-administration in humans. Biological Psychiatry, 65(10), 846–50. doi: 10.1016/j.biopsych.2009.01.021. 19249751

[ref39] Daniel R., Pollmann S. (2014). A universal role of the ventral striatum in reward-based learning: evidence from human studies. Neurobiology of Learning and Memory, 114, 90–100. doi: 10.1016/j.nlm.2014.05.002. 24825620 PMC4143465

[ref40] Delgado M.R. (2007). Reward-related responses in the human striatum. Annals of the New York Academy of Sciences, 1104, 70–88. doi: 10.1196/annals.1390.002. 17344522

[ref41] Denys D., Mantione M., Figee M., et al. (2010). Deep brain stimulation of the nucleus accumbens for treatment-refractory obsessive-compulsive disorder. Archives of General Psychiatry, 67(10), 1061–8. doi: 10.1001/archgenpsychiatry.2010.122. 20921122

[ref42] Dolan R.J. (2002). Emotion, cognition, and behavior. Science, 298(5596), 1191–4. doi: 10.1126/science.1076358. 12424363

[ref43] Doya K. (2000). Complementary roles of basal ganglia and cerebellum in learning and motor control. Current Opinion in Neurobiology, 10(6), 732–9. 11240282 10.1016/s0959-4388(00)00153-7

[ref44] Drapier D., Peron J., Leray E., et al. (2008). Emotion recognition impairment and apathy after subthalamic nucleus stimulation in Parkinson's disease have separate neural substrates. Neuropsychologia, 46(11), 2796–801. doi: 10.1016/j.neuropsychologia.2008.05.006. 18579165

[ref45] Dum R.P., Strick P.L. (2003). An unfolded map of the cerebellar dentate nucleus and its projections to the cerebral cortex. Journal of Neurophysiology, 89(1), 634–9. doi: 10.1152/jn.00626.2002. 12522208

[ref46] Eisinger R.S., Urdaneta M.E., Foote K.D., Okun M.S., Gunduz A. (2018). Non-motor characterization of the basal ganglia: evidence from human and non-human primate electrophysiology. Frontiers in Neuroscience, 12, 385–5. doi: 10.3389/fnins.2018.00385. 30026679 PMC6041403

[ref47] Escalona P.R., Early B., McDonald W.M., et al. (1993). Reduction of cerebellar volume in major depression: a controlled MRI study. Depression, 1(3), 156–8. doi: 10.1002/depr.3050010307.

[ref48] Fatemi S.H., Aldinger K.A., Ashwood P., et al. (2012). Consensus paper: pathological role of the cerebellum in autism. Cerebellum, 11(3), 777–807. doi: 10.1007/s12311-012-0355-9. 22370873 PMC3677555

[ref49] Fermin A.S., Yoshida T., Yoshimoto J., Ito M., Tanaka S.C., Doya K. (2016). Model-based action planning involves cortico-cerebellar and basal ganglia networks. Scientific Reports, 6, 31378. doi: 10.1038/srep31378. 27539554 PMC4990901

[ref50] Ferrari C., Oldrati V., Gallucci M., Vecchi T., Cattaneo Z. (2018). The role of the cerebellum in explicit and incidental processing of facial emotional expressions: a study with transcranial magnetic stimulation. NeuroImage, 169, 256–64. doi: 10.1016/j.neuroimage.2017.12.026. 29246845

[ref51] Ferrucci R., Giannicola G., Rosa M., et al. (2012). Cerebellum and processing of negative facial emotions: cerebellar transcranial DC stimulation specifically enhances the emotional recognition of facial anger and sadness. Cogn Emot, 26(5), 786–99. doi: 10.1080/02699931.2011.619520. 22077643 PMC4234053

[ref52] Friedman D.P., Aggleton J.P., Saunders R.C. (2002). Comparison of hippocampal, amygdala, and perirhinal projections to the nucleus accumbens: combined anterograde and retrograde tracing study in the macaque brain. The Journal of Comparative Neurology, 450(4), 345–65. doi: 10.1002/cne.10336. 12209848

[ref53] Frings M., Dimitrova A., Schorn C.F., et al. (2006). Cerebellar involvement in verb generation: an fMRI study. Neuroscience Letters, 409(1), 19–23. doi: 10.1016/j.neulet.2006.08.058. 17046160

[ref54] Grandjean D., Scherer K.R. (2008). Unpacking the cognitive architecture of emotion processes. Emotion, 8(3), 341–51. doi: 10.1037/1528-3542.8.3.341. 18540750

[ref55] Granziera C., Schmahmann J.D., Hadjikhani N., et al. (2009). Diffusion spectrum imaging shows the structural basis of functional cerebellar circuits in the human cerebellum in vivo. PLoS One, 4(4), e5101–1. doi: 10.1371/journal.pone.0005101. 19340289 PMC2659746

[ref56] Graybiel A.M. (1998). The basal ganglia and chunking of action repertoires. Neurobiology of Learning and Memory, 70(1), 119–36. doi: 10.1006/nlme.1998.3843. 9753592

[ref57] Graybiel A.M. (2008). Habits, rituals, and the evaluative brain. Annual Review of Neuroscience, 31, 359–87. doi: 10.1146/annurev.neuro.29.051605.112851. 18558860

[ref58] Guell X., Gabrieli J.D.E., Schmahmann J.D. (2018). Triple representation of language, working memory, social and emotion processing in the cerebellum: convergent evidence from task and seed-based resting-state fMRI analyses in a single large cohort. NeuroImage, 172, 437–49. doi: 10.1016/j.neuroimage.2018.01.082. 29408539 PMC5910233

[ref59] Gunaydin L.A., Kreitzer A.C. (2016). Cortico-basal ganglia circuit function in psychiatric disease. Annual Review of Physiology, 78, 327–50. doi: 10.1146/annurev-physiol-021115-105355. 26667072

[ref60] Habas C. (2010). Functional imaging of the deep cerebellar nuclei: a review. Cerebellum, 9(1), 22–8. doi: 10.1007/s12311-009-0119-3. 19513801

[ref61] Habas C. (2018). Research note: a resting-state, cerebello-amygdaloid intrinsically connected network. Cerebellum & Ataxias, 5(1), 4. doi: 10.1186/s40673-018-0083-0. 29468083 PMC5813397

[ref62] Habas C., Kamdar N., Nguyen D., et al. (2009). Distinct cerebellar contributions to intrinsic connectivity networks. The Journal of Neuroscience, 29(26), 8586–94. doi: 10.1523/jneurosci.1868-09.2009. 19571149 PMC2742620

[ref63] Haber S. (2008). Parallel and integrative processing through the basal ganglia reward circuit: lessons from addiction. Biological Psychiatry, 64(3), 173–4. doi: 10.1016/j.biopsych.2008.05.033. 18617023

[ref64] Haber S., Knutson B. (2010). The reward circuit: linking primate anatomy and human imaging. Neuropsychopharmacology, 35(1), 4–26. doi: 10.1038/npp.2009.129. 19812543 PMC3055449

[ref65] Halpern C.H., Rick J.H., Danish S.F., Grossman M., Baltuch G.H. (2009). Cognition following bilateral deep brain stimulation surgery of the subthalamic nucleus for Parkinson's disease. International Journal of Geriatric Psychiatry, 24(5), 443–51. doi: 10.1002/gps.2149. 19016252

[ref66] Haynes W.I.A., Haber S.N. (2013). The Organization of Prefrontal-Subthalamic Inputs in primates provides an anatomical substrate for both functional specificity and integration: implications for basal ganglia models and deep brain stimulation. The Journal of Neuroscience, 33(11), 4804–14. doi: 10.1523/jneurosci.4674-12.2013. 23486951 PMC3755746

[ref67] Heath R.G., Harper J.W. (1974). Ascending projections of the cerebellar fastigial nucleus to the hippocampus, amygdala, and other temporal lobe sites: evoked potential and histological studies in monkeys and cats. Experimental Neurology, 45(2), 268–87. 4422320 10.1016/0014-4886(74)90118-6

[ref68] Heimer L., Van Hoesen G.W. (2006). The limbic lobe and its output channels: implications for emotional functions and adaptive behavior. Neuroscience & Biobehavioral Reviews, 30(2), 126–47. doi: 10.1016/j.neubiorev.2005.06.006. 16183121

[ref69] Hernandez-Castillo C.R., Galvez V., Mercadillo R.E., et al. (2015). Functional connectivity changes related to cognitive and motor performance in spinocerebellar ataxia type 2. Movement Disorders, 30(10), 1391–9. doi: 10.1002/mds.26320. 26256273

[ref70] Herrera-Meza G., Aguirre-Manzo L., Coria-Avila G.A., et al. (2014). Beyond the basal ganglia: cFOS expression in the cerebellum in response to acute and chronic dopaminergic alterations. Neuroscience, 267, 219–31. doi: 10.1016/j.neuroscience.2014.02.046. 24631673

[ref71] Hoshi E., Tremblay L., Feger J., Carras P.L., Strick P.L. (2005). The cerebellum communicates with the basal ganglia. Nature Neuroscience, 8(11), 1491–3. doi: 10.1038/nn1544. 16205719

[ref72] Ikai Y., Takada M., Shinonaga Y., Mizuno N. (1992). Dopaminergic and non-dopaminergic neurons in the ventral tegmental area of the rat project, respectively, to the cerebellar cortex and deep cerebellar nuclei. Neuroscience, 51(3), 719–28. doi: 10.1016/0306-4522(92)90310-x. 1362601

[ref73] Ikemoto S., Yang C., Tan A. (2015). Basal ganglia circuit loops, dopamine and motivation: a review and enquiry. Behavioural Brain Research, 290, 17–31. doi: 10.1016/j.bbr.2015.04.018. 25907747 PMC4447603

[ref74] Irmen F., Huebl J., Schroll H., et al. (2017). Subthalamic nucleus stimulation impairs emotional conflict adaptation in Parkinson's disease. Social Cognitive and Affective Neuroscience, 12(10), 1594–604. doi: 10.1093/scan/nsx090. 28985419 PMC5647801

[ref75] Jin X., Tecuapetla F., Costa R.M. (2014). Basal ganglia subcircuits distinctively encode the parsing and concatenation of action sequences. Nature Neuroscience, 17, 423. doi: 10.1038/nn.3632. 24464039 PMC3955116

[ref76] Jueptner M., Weiller C. (1998). A review of differences between basal ganglia and cerebellar control of movements as revealed by functional imaging studies. Brain, 121(Pt 8), 1437–49. 9712006 10.1093/brain/121.8.1437

[ref77] Keeler J.F., Pretsell D.O., Robbins T.W. (2014). Functional implications of dopamine D1 vs. D2 receptors: a ‘prepare and select’ model of the striatal direct vs. indirect pathways. Neuroscience, 282, 156–75. doi: 10.1016/j.neuroscience.2014.07.021. 25062777

[ref78] Kelly R.M., Strick P.L. (2003). Cerebellar loops with motor cortex and prefrontal cortex of a nonhuman primate. The Journal of Neuroscience, 23(23), 8432–44. 12968006 10.1523/JNEUROSCI.23-23-08432.2003 PMC6740694

[ref79] Kim S.G., Ugurbil K., Strick P.L. (1994). Activation of a cerebellar output nucleus during cognitive processing. Science, 265(5174), 949–51. 8052851 10.1126/science.8052851

[ref80] Knutson B., Taylor J., Kaufman M., Peterson R., Glover G. (2005). Distributed neural representation of expected value. The Journal of Neuroscience, 25(19), 4806–12. doi: 10.1523/jneurosci.0642-05.2005. 15888656 PMC6724773

[ref81] Kostadinov D., Beau M., Pozo M.B., Hausser M. (2019). Predictive and reactive reward signals conveyed by climbing fiber inputs to cerebellar Purkinje cells. Nature Neuroscience, 22(6), 950–62. doi: 10.1038/s41593-019-0381-8. 31036947 PMC7612392

[ref82] Koziol L.F., Budding D., Andreasen N., et al. (2014). Consensus paper: the Cerebellum's role in movement and cognition. The Cerebellum, 13(1), 151–77. doi: 10.1007/s12311-013-0511-x. 23996631 PMC4089997

[ref83] Krack P., Hariz M.I., Baunez C., Guridi J., Obeso J.A. (2010). Deep brain stimulation: from neurology to psychiatry? Trends in Neurosciences, 33(10), 474–84. doi: 10.1016/j.tins.2010.07.002. 20832128

[ref84] Kravitz A.V., Tye L.D., Kreitzer A.C. (2012). Distinct roles for direct and indirect pathway striatal neurons in reinforcement. Nature Neuroscience, 15(6), 816–8. doi: 10.1038/nn.3100. 22544310 PMC3410042

[ref85] Kuhn A.A., Hariz M.I., Silberstein P., et al. (2005). Activation of the subthalamic region during emotional processing in Parkinson disease. Neurology, 65(5), 707–13. doi: 10.1212/01.wnl.0000174438.78399.bc. 16157903

[ref86] Kuper M., Dimitrova A., Thurling M., et al. (2011). Evidence for a motor and a non-motor domain in the human dentate nucleus--an fMRI study. NeuroImage, 54(4), 2612–22. doi: 10.1016/j.neuroimage.2010.11.028. 21081171

[ref87] Lambert C., Zrinzo L., Nagy Z., et al. (2012). Confirmation of functional zones within the human subthalamic nucleus: patterns of connectivity and sub-parcellation using diffusion weighted imaging. NeuroImage, 60(1), 83–94. doi: 10.1016/j.neuroimage.2011.11.082. 22173294 PMC3315017

[ref88] Lanciego J.L., Luquin N., Obeso J.A. (2012). Functional neuroanatomy of the basal ganglia. Cold Spring Harbor Perspectives in Medicine, 2(12), a009621. doi: 10.1101/cshperspect.a009621. 23071379 PMC3543080

[ref89] Le Jeune F., Péron J., Biseul I., et al. (2008). Subthalamic nucleus stimulation affects orbitofrontal cortex in facial emotion recognition: a PET study. Brain, 131(Pt 6), 1599–608. doi:10.1093/brain/awn084 18490359 PMC2408938

[ref90] Lee G.P., Meador K.J., Loring D.W., et al. (2004). Neural substrates of emotion as revealed by functional magnetic resonance imaging. Cognitive and Behavioral Neurology, 17(1), 9–17. 15209221 10.1097/00146965-200403000-00002

[ref91] Leggio M., Olivito G. (2018). Topography of the cerebellum in relation to social brain regions and emotions. Handbook of Clinical Neurology, 154, 71–84. doi: 10.1016/b978-0-444-63956-1.00005-9. 29903453

[ref92] Leroi I., O'Hearn E., Marsh L., et al. (2002). Psychopathology in patients with degenerative cerebellar diseases: a comparison to Huntington's disease. The American Journal of Psychiatry, 159(8), 1306–14. doi: 10.1176/appi.ajp.159.8.1306. 12153822

[ref93] Lesage E., Morgan B.E., Olson A.C., Meyer A.S., Miall R.C. (2012). Cerebellar rTMS disrupts predictive language processing. Current Biology, 22(18), R794–5. doi: 10.1016/j.cub.2012.07.006. 23017990 PMC3459089

[ref94] Levisohn L., Cronin-Golomb A., Schmahmann J.D. (2000). Neuropsychological consequences of cerebellar tumour resection in children: cerebellar cognitive affective syndrome in a paediatric population. Brain, 123(Pt 5), 1041–50. doi: 10.1093/brain/123.5.1041. 10775548

[ref95] Luijten M., Schellekens A.F., Kuhn S., Machielse M.W., Sescousse G. (2017). Disruption of reward processing in addiction: an image-based meta-analysis of functional magnetic resonance imaging studies. JAMA Psychiatry, 74(4), 387–98. doi: 10.1001/jamapsychiatry.2016.3084. 28146248

[ref96] Lupo M., Troisi E., Chiricozzi F.R., Clausi S., Molinari M., Leggio M. (2015). Inability to process negative emotions in cerebellar damage: a functional Transcranial Doppler Sonographic study. The Cerebellum, 14(6), 663–9. doi: 10.1007/s12311-015-0662-z. 25784354

[ref97] Lupo M., Olivito G., Siciliano L., et al. (2018). Development of a psychiatric disorder linked to cerebellar lesions. Cerebellum, 17(4), 438–46. doi: 10.1007/s12311-018-0926-5. 29460204

[ref98] Makris N., Oscar-Berman M., Jaffin S.K., et al. (2008). Decreased volume of the brain reward system in alcoholism. Biological Psychiatry, 64(3), 192–202. doi: 10.1016/j.biopsych.2008.01.018. 18374900 PMC2572710

[ref99] Marek S., Siegel J.S., Gordon E.M., et al. (2018). Spatial and temporal Organization of the Individual Human Cerebellum. Neuron, 100(4), 977–993.e977. doi: 10.1016/j.neuron.2018.10.010. 30473014 PMC6351081

[ref100] Markram H., Lübke J., Frotscher M., Sakmann B. (1997). Regulation of synaptic efficacy by coincidence of postsynaptic APs and EPSPs. Science, 275(5297), 213. doi: 10.1126/science.275.5297.213. 8985014

[ref101] Mathai A., Smith Y. (2011). The corticostriatal and corticosubthalamic pathways: two entries, one target. So what? Frontiers in Systems Neuroscience, 5, 64. doi: 10.3389/fnsys.2011.00064. 21866224 PMC3149683

[ref102] Mehanna R., Bajwa J.A., Fernandez H., Wagle Shukla A.A. (2017). Cognitive impact of deep brain stimulation on Parkinson's disease patients. Parkinsons Disease, 2017, 3085140. doi: 10.1155/2017/3085140. PMC5735627 29359065

[ref103] Middleton F.A., Strick P.L. (1994). Anatomical evidence for cerebellar and basal ganglia involvement in higher cognitive function. Science, 266(5184), 458–61. 7939688 10.1126/science.7939688

[ref104] Middleton F.A., Strick P.L. (2000). Basal ganglia and cerebellar loops: motor and cognitive circuits. Brain Research. Brain Research Reviews, 31(2–3), 236–50. 10719151 10.1016/s0165-0173(99)00040-5

[ref105] Milardi D., Arrigo A., Anastasi G., et al. (2016). Extensive direct subcortical cerebellum-basal ganglia connections in human brain as revealed by constrained spherical Deconvolution Tractography. Frontiers in Neuroanatomy, 10(29), 1–10. doi: 10.3389/fnana.2016.00029. 27047348 PMC4796021

[ref106] Miller E.M., Shankar M.U., Knutson B., McClure S.M. (2014). Dissociating motivation from reward in human striatal activity. Journal of Cognitive Neuroscience, 26(5), 1075–84. doi: 10.1162/jocn_a_00535. 24345173 PMC5808846

[ref107] Mink J.W. (1996). The basal ganglia: focused selection and inhibition of competing motor programs. Progress in Neurobiology, 50(4), 381–425. 9004351 10.1016/s0301-0082(96)00042-1

[ref108] Moum S.J., Price C.C., Limotai N., et al. (2012). Effects of STN and GPi deep brain stimulation on impulse control disorders and dopamine dysregulation syndrome. PLoS One, 7(1), e29768. doi: 10.1371/journal.pone.0029768. 22295068 PMC3266249

[ref109] Nambu A., Tokuno H., Takada M. (2002). Functional significance of the cortico–subthalamo–pallidal ‘hyperdirect’ pathway. Neuroscience Research, 43(2), 111–7. doi: 10.1016/S0168-0102(02)00027-5. 12067746

[ref110] Olivito G., Cercignani M., Lupo M., et al. (2017a). Neural substrates of motor and cognitive dysfunctions in SCA2 patients: a network based statistics analysis. Neuroimage Clinical, 14, 719–25. doi: 10.1016/j.nicl.2017.03.009. 28393013 PMC5377430

[ref111] Olivito G., Dayan M., Battistoni V., et al. (2017b). Bilateral effects of unilateral cerebellar lesions as detected by voxel based morphometry and diffusion imaging. PLoS One, 12(7), e0180439. doi: 10.1371/journal.pone.0180439. 28692678 PMC5503258

[ref112] Olivito G., Lupo M., Iacobacci C., et al. (2018). Structural cerebellar correlates of cognitive functions in spinocerebellar ataxia type 2. Journal of Neurology, 265(3), 597–606. doi: 10.1007/s00415-018-8738-6. 29356974

[ref113] Ory S., Le Jeune F., Haegelen C., et al. (2017). Pre-frontal-insular-cerebellar modifications correlate with disgust feeling blunting after subthalamic stimulation: a positron emission tomography study in Parkinson's disease. Journal of Neuropsychology, 11(3), 378–95. doi: 10.1111/jnp.12094. 26670087

[ref114] Parent A., Hazrati L.N. (1995a). Functional anatomy of the basal ganglia. I. The cortico-basal ganglia-thalamo-cortical loop. Brain Research. Brain Research Reviews, 20(1), 91–127. 7711769 10.1016/0165-0173(94)00007-c

[ref115] Parent A., Hazrati L.N. (1995b). Functional anatomy of the basal ganglia. II. The place of subthalamic nucleus and external pallidum in basal ganglia circuitry. Brain Research. Brain Research Reviews, 20(1), 128–54. 7711765 10.1016/0165-0173(94)00008-d

[ref116] Paul K., Pourtois G., Harmon-Jones E. (2020). Modulatory effects of positive mood and approach motivation on reward processing: two sides of the same coin? Cognitive, Affective, & Behavioral Neuroscience, 20, 236–49. doi: 10.3758/s13415-019-00764-6. 32043206

[ref117] Pelzer E.A., Hintzen A., Goldau M., von Cramon D.Y., Timmermann L., Tittgemeyer M. (2013). Cerebellar networks with basal ganglia: feasibility for tracking cerebello-pallidal and subthalamo-cerebellar projections in the human brain. The European Journal of Neuroscience, 38(8), 3106–14. doi: 10.1111/ejn.12314. 23879686

[ref118] Pelzer E.A., Melzer C., Timmermann L., von Cramon D.Y., Tittgemeyer M. (2017). Basal ganglia and cerebellar interconnectivity within the human thalamus. Brain Structure and Function, 222(1), 381–92. doi: 10.1007/s00429-016-1223-z. 27089884 PMC5225161

[ref119] Péron J., Grandjean D., Le Jeune F., et al. (2010). Recognition of emotional prosody is altered after subthalamic nucleus deep brain stimulation in Parkinson's disease. Neuropsychologia, 48(4), 1053–62. doi: 10.1016/j.neuropsychologia.2009.12.003. 20005239

[ref120] Péron J., Fruhholz S., Verin M., Grandjean D. (2013). Subthalamic nucleus: a key structure for emotional component synchronization in humans. Neuroscience and Biobehavioral Reviews, 37(3), 358–73. doi: 10.1016/j.neubiorev.2013.01.001. 23318227

[ref121] Péron J., Frühholz S., Ceravolo L., Grandjean D. (2016). Structural and functional connectivity of the subthalamic nucleus during vocal emotion decoding. Social Cognitive and Affective Neuroscience, 11(2), 349–56. doi: 10.1093/scan/nsv118. 26400857 PMC4733346

[ref122] Péron J., Renaud O., Haegelen C., et al. (2017). Vocal emotion decoding in the subthalamic nucleus: an intracranial ERP study in Parkinson’s disease. Brain and Language, 168, 1–11. doi: 10.1016/j.bandl.2016.12.003. 28088666

[ref123] Peterburs J., Desmond J.E. (2016). The role of the human cerebellum in performance monitoring. Current Opinion in Neurobiology, 40, 38–44. doi: 10.1016/j.conb.2016.06.011. 27372055 PMC5056810

[ref124] Peterburs J., Hofmann D., Becker M.P.I., Nitsch A.M., Miltner W.H.R., Straube T. (2018). The role of the cerebellum for feedback processing and behavioral switching in a reversal-learning task. Brain and Cognition, 125, 142–8. doi: 10.1016/j.bandc.2018.07.001. 29990704

[ref125] Petersen S.E., Fox P.T., Posner M.I., Mintun M., Raichle M.E. (1989). Positron emission tomographic studies of the processing of singe words. Journal of Cognitive Neuroscience, 1(2), 153–70. doi: 10.1162/jocn.1989.1.2.153. 23968463

[ref126] Pidoux L., Le Blanc P., Levenes C., Leblois A. (2018). A subcortical circuit linking the cerebellum to the basal ganglia engaged in vocal learning. eLife, 7. doi: 10.7554/eLife.32167. PMC6112851 30044222

[ref127] Pine A., Sadeh N., Ben-Yakov A., Dudai Y., Mendelsohn A. (2018). Knowledge acquisition is governed by striatal prediction errors. Nature Communications, 9(1), 1673. doi: 10.1038/s41467-018-03992-5. PMC5919975 29700377

[ref128] Pleger B., Timmann D. (2018). The role of the human cerebellum in linguistic prediction, word generation and verbal working memory: evidence from brain imaging, non-invasive cerebellar stimulation and lesion studies. Neuropsychologia, 115, 204–10. doi: 10.1016/j.neuropsychologia.2018.03.012. 29530801

[ref129] Popa L.S., Ebner T.J. (2019). Cerebellum, predictions and errors. Frontiers in Cellular Neuroscience, 12, 524–4. doi: 10.3389/fncel.2018.00524. 30697149 PMC6340992

[ref130] Ramnani N., Elliott R., Athwal B.S., Passingham R.E. (2004). Prediction error for free monetary reward in the human prefrontal cortex. NeuroImage, 23(3), 777–86. doi: 10.1016/j.neuroimage.2004.07.028. 15528079

[ref131] Robbins T.W., Everitt B.J. (1996). Neurobehavioural mechanisms of reward and motivation. Current Opinion in Neurobiology, 6(2), 228–36. doi: 10.1016/s0959-4388(96)80077-8. 8725965

[ref132] Robinson O.J., Cools R., Carlisi C.O., Sahakian B.J., Drevets W.C. (2012). Ventral striatum response during reward and punishment reversal learning in unmedicated major depressive disorder. The American Journal of Psychiatry, 169(2), 152–9. doi: 10.1176/appi.ajp.2011.11010137. 22420038 PMC5648982

[ref133] Rossi P.J., Gunduz A., Okun M.S. (2015). The subthalamic nucleus, limbic function, and impulse control. Neuropsychology Review, 25(4), 398–410. doi: 10.1007/s11065-015-9306-9. 26577509 PMC4792181

[ref134] Rudebeck P.H., Rich E.L. (2018). Orbitofrontal cortex. Current Biology, 28(18), R1083–r1088. doi: 10.1016/j.cub.2018.07.018. 30253144 PMC9253859

[ref135] Sacchetti B., Baldi E., Lorenzini C.A., Bucherelli C. (2002). Cerebellar role in fear-conditioning consolidation. Proceedings of the National Academy of Sciences of the United States of America, 99(12), 8406–11. doi: 10.1073/pnas.112660399. 12034877 PMC123080

[ref136] Sander D., Grafman J., Zalla T. (2003). The human amygdala: an evolved system for relevance detection. Reviews in the Neurosciences, 14(4), 303–16. doi: 10.1515/revneuro.2003.14.4.303. 14640318

[ref137] Sander D., Grandjean D., Scherer K.R. (2018). An appraisal-driven componential approach to the emotional brain. Emotion Review, 10(3), 219–31. doi: 10.1177/1754073918765653.

[ref138] Sang L., Qin W., Liu Y., et al. (2012). Resting-state functional connectivity of the vermal and hemispheric subregions of the cerebellum with both the cerebral cortical networks and subcortical structures. NeuroImage, 61(4), 1213–25. doi: 10.1016/j.neuroimage.2012.04.011. 22525876

[ref139] Scherer K.R. (2009). The dynamic architecture of emotion: evidence for the component process model. Cognition and Emotion, 23(7), 1307–51. doi: 10.1080/02699930902928969.

[ref140] Schienle A., Scharmuller W. (2013). Cerebellar activity and connectivity during the experience of disgust and happiness. Neuroscience, 246, 375–81. doi: 10.1016/j.neuroscience.2013.04.048. 23639880

[ref141] Schirmer A., Kotz S.A. (2006). Beyond the right hemisphere: brain mechanisms mediating vocal emotional processing. Trends in Cognitive Sciences, 10(1), 24–30. doi: 10.1016/j.tics.2005.11.009. 16321562

[ref142] Schmahmann J.D. (1991). An emerging concept: the cerebellar contribution to higher function. Archives of Neurology, 48(11), 1178–87. doi: 10.1001/archneur.1991.00530230086029. 1953406

[ref143] Schmahmann J.D. (2019). The cerebellum and cognition. Neuroscience Letters, 688, 62–75. doi: 10.1016/j.neulet.2018.07.005. 29997061

[ref144] Schmahmann J.D., Sherman J.C. (1997). Cerebellar cognitive affective syndrome In: Schmahmann J.D., editor. Int Rev Neurobiol, Vol. 41, San Diego, CA: Academic Press, pp. 433–40. 10.1016/s0074-7742(08)60363-3 9378601

[ref145] Schmahmann J.D., Sherman J.C. (1998). The cerebellar cognitive affective syndrome. Brain, 121(Pt 4), 561–79. doi: 10.1093/brain/121.4.561. 9577385

[ref146] Schmahmann J.D., Weilburg J.B., Sherman J.C. (2007). The neuropsychiatry of the cerebellum — insights from the clinic. The Cerebellum, 6(3), 254–67. doi: 10.1080/14734220701490995. 17786822

[ref147] Schraa-Tam C.K., Rietdijk W.J., Verbeke W.J., et al. (2012). fMRI activities in the emotional cerebellum: a preference for negative stimuli and goal-directed behavior. Cerebellum, 11(1), 233–45. doi: 10.1007/s12311-011-0301-2. 21761197 PMC3311856

[ref148] Schroeder U., Kuehler A., Hennenlotter A., et al. (2004). Facial expression recognition and subthalamic nucleus stimulation. Journal of Neurology, Neurosurgery and Psychiatry, 75(4), 648. doi: 10.1136/jnnp.2003.019794. PMC1739017 15026519

[ref149] Schultz W., Dayan P., Montague P.R. (1997). A neural substrate of prediction and reward. Science, 275(5306), 1593–9. doi: 10.1126/science.275.5306.1593. 9054347

[ref150] Schultz W., Tremblay L., Hollerman J.R. (2000). Reward processing in primate orbitofrontal cortex and basal ganglia. Cerebral Cortex, 10(3), 272–84. doi: 10.1093/cercor/10.3.272. 10731222

[ref151] Schutter D.J., van Honk J. (2009). The cerebellum in emotion regulation: a repetitive transcranial magnetic stimulation study. Cerebellum, 8(1), 28–34. doi: 10.1007/s12311-008-0056-6. 18855096

[ref152] Schutter D.J., Enter D., Hoppenbrouwers S.S. (2009). High-frequency repetitive transcranial magnetic stimulation to the cerebellum and implicit processing of happy facial expressions. Journal of Psychiatry & Neuroscience, 34(1), 60–5. 19125214 PMC2612080

[ref153] Schutter D.J., Koolschijn P.C., Peper J.S., Crone E.A. (2012). The cerebellum link to neuroticism: a volumetric MRI association study in healthy volunteers. PLoS One, 7(5), e37252. doi: 10.1371/journal.pone.0037252. 22615955 PMC3355107

[ref154] Scott J.A., Schumann C.M., Goodlin-Jones B.L., Amaral D.G. (2009). A comprehensive volumetric analysis of the cerebellum in children and adolescents with autism spectrum disorder. Autism Research, 2(5), 246–57. doi: 10.1002/aur.97. 19885834 PMC2999464

[ref155] Shigemune Y., Tsukiura T., Kambara T., Kawashima R. (2013). Remembering with gains and losses: effects of monetary reward and punishment on successful encoding activation of source memories. Cerebral Cortex, 24(5), 1319–31. doi: 10.1093/cercor/bhs415. 23314939 PMC3977621

[ref156] Sieger T., Serranova T., Ruzicka F., et al. (2015). Distinct populations of neurons respond to emotional valence and arousal in the human subthalamic nucleus. Proceedings of the National Academy of Sciences of the United States of America, 112(10), 3116–21. doi: 10.1073/pnas.1410709112. 25713375 PMC4364224

[ref157] Simonyan K. (2019). Recent advances in understanding the role of the basal ganglia. F1000Res, 8. doi: 10.12688/f1000research.16524.1. PMC6354324 30755797

[ref158] Sokolov A.A., Miall R.C., Ivry R.B. (2017). The cerebellum: adaptive prediction for movement and cognition. Trends in Cognitive Sciences, 21(5), 313–32. doi: 10.1016/j.tics.2017.02.005. 28385461 PMC5477675

[ref159] Sokolovsky N., Cook A., Hunt H., Giunti P., Cipolotti L. (2010). A preliminary characterisation of cognition and social cognition in Spinocerebellar ataxia types 2, 1, and 7. Behavioural Neurology, 23(1–2), 17–29. doi: 10.3233/ben-2010-0270. 20714058 PMC5434399

[ref160] Stirnimann N., N'Diaye K., Jeune F.L., et al. (2018). Hemispheric specialization of the basal ganglia during vocal emotion decoding: evidence from asymmetric Parkinson's disease and (18)FDG PET. Neuropsychologia, 119, 1–11. doi: 10.1016/j.neuropsychologia.2018.07.023. 30040955

[ref161] Stoodley C.J., Schmahmann J.D. (2009). Functional topography in the human cerebellum: a meta-analysis of neuroimaging studies. NeuroImage, 44(2), 489–501. doi: 10.1016/j.neuroimage.2008.08.039. 18835452

[ref162] Stoodley C.J., Schmahmann J.D. (2018). Chapter 4 - Functional topography of the human cerebellum In: Manto M., Huisman T.A.G.M., editors. Handb Clin Neurol, Vol. 154, Cambridge, MA: Elsevier, pp. 59–70. 10.1016/B978-0-444-63956-1.00004-7 29903452

[ref163] Strata P. (2015). The emotional cerebellum. Cerebellum, 14(5), 570–7. doi: 10.1007/s12311-015-0649-9. 25626523

[ref164] Subramanian K., Brandenburg C., Orsati F., Soghomonian J.J., Hussman J.P., Blatt G.J. (2017). Basal ganglia and autism - a translational perspective. Autism Research, 10(11), 1751–75. doi: 10.1002/aur.1837. 28730641

[ref165] Supple W.F. Jr., Leaton R.N., Fanselow M.S. (1987). Effects of cerebellar vermal lesions on species-specific fear responses, neophobia, and taste-aversion learning in rats. Physiology & Behavior, 39(5), 579–86. doi: 10.1016/0031-9384(87)90156-9. 3588702

[ref166] Tanaka S.C., Doya K., Okada G., Ueda K., Okamoto Y., Yamawaki S. (2004). Prediction of immediate and future rewards differentially recruits cortico-basal ganglia loops. Nature Neuroscience, 7, 887. doi: 10.1038/nn1279 https://www.nature.com/articles/nn1279#supplementary-information. 15235607

[ref167] Taylor J.A., Ivry R.B. (2014). Cerebellar and prefrontal cortex contributions to adaptation, strategies, and reinforcement learning. Progress in Brain Research, 210, 217–53. doi: 10.1016/B978-0-444-63356-9.00009-1. 24916295 PMC4118688

[ref168] Temel Y., Blokland A., Steinbusch H.W., Visser-Vandewalle V. (2005). The functional role of the subthalamic nucleus in cognitive and limbic circuits. Progress in Neurobiology, 76(6), 393–413. doi: 10.1016/j.pneurobio.2005.09.005. 16249050

[ref169] Temel Y., Kessels A., Tan S., Topdag A., Boon P., Visser-Vandewalle V. (2006). Behavioural changes after bilateral subthalamic stimulation in advanced Parkinson disease: a systematic review. Parkinsonism & Related Disorders, 12(5), 265–72. doi: 10.1016/j.parkreldis.2006.01.004. 16621661

[ref170] Thielscher A., Pessoa L. (2007). Neural correlates of perceptual choice and decision making during fear-disgust discrimination. The Journal of Neuroscience, 27(11), 2908–17. doi: 10.1523/jneurosci.3024-06.2007. 17360913 PMC6672587

[ref171] Thomasson M., Saj A., Benis D., Grandjean D., Assal F., Peron J. (2019). Cerebellar contribution to vocal emotion decoding: insights from stroke and neuroimaging. Neuropsychologia, 132, 107141. doi: 10.1016/j.neuropsychologia.2019.107141. 31306617

[ref172] Turner B.M., Paradiso S., Marvel C.L., et al. (2007). The cerebellum and emotional experience. Neuropsychologia, 45(6), 1331–41. doi: 10.1016/j.neuropsychologia.2006.09.023. 17123557 PMC1868674

[ref173] Ubl B., Kuehner C., Kirsch P., Ruttorf M., Diener C., Flor H. (2015). Altered neural reward and loss processing and prediction error signalling in depression. Social Cognitive and Affective Neuroscience, 10(8), 1102–12. doi: 10.1093/scan/nsu158. 25567763 PMC4526479

[ref174] Van Overwalle F., Baetens K., Marien P., Vandekerckhove M. (2014). Social cognition and the cerebellum: a meta-analysis of over 350 fMRI studies. NeuroImage, 86, 554–72. doi: 10.1016/j.neuroimage.2013.09.033. 24076206

[ref175] Vicente S., Biseul I., Peron J., et al. (2009). Subthalamic nucleus stimulation affects subjective emotional experience in Parkinson's disease patients. Neuropsychologia, 47(8–9), 1928–37. doi: 10.1016/j.neuropsychologia.2009.03.003. 19428425

[ref176] Volkmann J., Allert N., Voges J., Weiss P.H., Freund H.J., Sturm V. (2001). Safety and efficacy of pallidal or subthalamic nucleus stimulation in advanced PD. Neurology, 56(4), 548–51. doi: 10.1212/wnl.56.4.548. 11222806

[ref177] Volkmann J., Daniels C., Witt K. (2010). Neuropsychiatric effects of subthalamic neurostimulation in Parkinson disease. Nature Reviews Neurology, 6(9), 487–98. doi: 10.1038/nrneurol.2010.111. 20680036

[ref178] Vuilleumier P. (2005). How brains beware: neural mechanisms of emotional attention. Trends in Cognitive Sciences, 9(12), 585–94. doi: 10.1016/j.tics.2005.10.011. 16289871

[ref179] Wagenbreth C., Kuehne M., Heinze H.J., Zaehle T. (2019). Deep brain stimulation of the subthalamic nucleus influences facial emotion recognition in patients with Parkinson's disease: a review. Frontiers in Psychology, 10, 2638. doi: 10.3389/fpsyg.2019.02638. 31849760 PMC6901782

[ref180] Wagner M.J., Kim T.H., Savall J., Schnitzer M.J., Luo L. (2017). Cerebellar granule cells encode the expectation of reward. Nature, 544(7648), 96–100. doi: 10.1038/nature21726. 28321129 PMC5532014

[ref181] Wang D., Buckner R.L., Liu H. (2013). Cerebellar asymmetry and its relation to cerebral asymmetry estimated by intrinsic functional connectivity. Journal of Neurophysiology, 109(1), 46–57. doi: 10.1152/jn.00598.2012. 23076113 PMC3545158

[ref182] Wang K.S., Smith D.V., Delgado M.R. (2016). Using fMRI to study reward processing in humans: past, present, and future. Journal of Neurophysiology, 115(3), 1664–78. doi: 10.1152/jn.00333.2015. 26740530 PMC4808130

[ref183] Watson T., Koutsikou S., Cerminara N., et al. (2013). The olivo-cerebellar system and its relationship to survival circuits. Frontiers in Neural Circuits, 7(72), 1–7. doi: 10.3389/fncir.2013.00072. 23630468 PMC3632748

[ref184] Whitney E.R., Kemper T.L., Bauman M.L., Rosene D.L., Blatt G.J. (2008). Cerebellar Purkinje cells are reduced in a subpopulation of autistic brains: a stereological experiment using calbindin-D28k. Cerebellum, 7(3), 406–16. doi: 10.1007/s12311-008-0043-y. 18587625

[ref185] Wojtecki L., Elben S., Vesper J., Schnitzler A. (2017). The rhythm of the executive gate of speech: subthalamic low-frequency oscillations increase during verbal generation. European Journal of Neuroscience, 45(9), 1200–11. doi: 10.1111/ejn.13429. 27718535

[ref186] Wolpert D.M., Miall R.C., Kawato M. (1998). Internal models in the cerebellum. Trends in Cognitive Sciences, 2(9), 338–47. doi: 10.1016/S1364-6613(98)01221-2. 21227230

[ref187] Wu T., Hallett M. (2005). A functional MRI study of automatic movements in patients with Parkinson's disease. Brain, 128(Pt 10), 2250–9. doi:10.1093/brain/awh569 15958505

[ref188] Wu B., Han L., Sun B.M., Hu X.W., Wang X.P. (2014). Influence of deep brain stimulation of the subthalamic nucleus on cognitive function in patients with Parkinson's disease. Neuroscience Bulletin, 30(1), 153–61. doi: 10.1007/s12264-013-1389-9. 24338433 PMC5562574

[ref189] Xiao L., Bornmann C., Hatstatt-Burklé L., Scheiffele P. (2018). Regulation of striatal cells and goal-directed behavior by cerebellar outputs. Nature Communications, 9(1), 3133. doi: 10.1038/s41467-018-05565-y. PMC6081479 30087345

[ref190] Yin H.H., Knowlton B.J., Balleine B.W. (2004). Lesions of dorsolateral striatum preserve outcome expectancy but disrupt habit formation in instrumental learning. European Journal of Neuroscience, 19(1), 181–9. doi: 10.1111/j.1460-9568.2004.03095.x. 14750976

[ref191] Yin H.H., Ostlund S.B., Balleine B.W. (2008). Reward-guided learning beyond dopamine in the nucleus accumbens: the integrative functions of cortico-basal ganglia networks. The European Journal of Neuroscience, 28(8), 1437–48. doi: 10.1111/j.1460-9568.2008.06422.x. 18793321 PMC2756656

[ref192] Yu H., Sternad D., Corcos D.M., Vaillancourt D.E. (2007). Role of hyperactive cerebellum and motor cortex in Parkinson's disease. NeuroImage, 35(1), 222–33. doi: 10.1016/j.neuroimage.2006.11.047. 17223579 PMC1853309

[ref193] Zangaglia R., Pacchetti C., Pasotti C., et al. (2009). Deep brain stimulation and cognitive functions in Parkinson's disease: a three-year controlled study. Movement Disorders, 24(11), 1621–8. doi: 10.1002/mds.22603. 19514093

[ref194] Zhu L., Sacco T., Strata P., Sacchetti B. (2011). Basolateral amygdala inactivation impairs learning-induced long-term potentiation in the cerebellar cortex. PLoS One, 6(1), e16673. doi: 10.1371/journal.pone.0016673. 21304962 PMC3031621

